# Tailoring BoNT A treatment and assessment across recovery stages after stroke: a report of four clinical cases

**DOI:** 10.25122/jml-2025-0151

**Published:** 2025-10

**Authors:** Miruna Săndulescu, Delia Cinteză, Daniel Ionuţ Răducan, Daniela Poenaru, Claudia-Gabriela Potcovaru, Horia Păunescu, Oana Andreia Coman

**Affiliations:** 1 Department of Physical and Rehabilitation Medicine, Carol Davila University of Medicine and Pharmacy, Bucharest, Romania; 2 Vasile Goldiş Western University, Arad, Romania; 3 Department of Pharmacology and Pharmacotherapy, Carol Davila University of Medicine and Pharmacy, Bucharest, Romania

**Keywords:** post-stroke spasticity, abobotulinum toxin, functional assessment, functional outcome

## Abstract

Achieving effective spasticity management in post-stroke patients remains a significant therapeutic challenge. It requires the anticipation and management of multiple potential complications through a complex, individualized therapeutic approach. The therapeutic goals in stroke-related spasticity vary considerably depending on the intensity and duration of spasticity, as well as the degree of motor control in the affected limb segments. This study presents four clinical case reports involving patients with post-stroke spasticity ranging from grade 1+ to 4 on the Modified Ashworth Scale (MAS), each exhibiting a distinct temporal profile of symptom progression and levels of motor control in affected limbs. All patients received conservative rehabilitation therapy in conjunction with botulinum toxin (BoNT-A) administration. Spasticity assessment is essential for evaluating treatment efficacy and for planning and refining rehabilitation strategies. Employing case-appropriate functional clinical scales facilitates dynamic assessment and quantification of motor deficits, thereby enabling precise definition and ongoing monitoring of therapeutic goals. Given the heterogeneous functional status of patients with post-stroke spasticity, therapeutic objectives and evaluation strategies must be tailored accordingly. BoNT-A therapy necessitates a patient-specific approach concerning dosing and injection intervals. Repeated BoNT-A treatment in cases of severe spasticity produced sustained reductions in limb pain and mitigated periarticular tissue damage. In patients with mild spasticity and preserved motor function, functionality reached substantial recovery, as reflected in outcomes from appropriately selected functional measures, with injections spaced at intervals exceeding three months and employing progressively lower doses.

## Introduction

The clinical and functional profile of patients with post-stroke spasticity is highly variable, prompting the need for individualized assessment protocols and rehabilitation objectives. The availability of numerous standardized functional scales for stroke assessment underscores the importance of selecting those most relevant to the patient’s clinical status. Therefore, the clinical team must have a thorough understanding of these tools, including their feasibility and relevance, to effectively monitor the patient’s progress in alignment with therapeutic objectives.

Spasticity is characterized by increased muscle tone resulting from stretch reflex hyperexcitability and is a hallmark of upper motor neuron syndrome (UMNS) following cerebral or spinal injury [1-3]. It is a sensorimotor disorder caused by central nervous system (CNS) damage and is manifested through sustained or intermittent involuntary muscle contractions [4,5]. The impact of spasticity extends beyond motor deficits, impairing both active limb use (e.g., task execution) and passive limb function (e.g., hygiene and positioning). It is often associated with pain, psychological distress, and disability, potentially leading to complications such as reduced mobility, impaired self-care, diminished self-esteem, pressure ulcers, and increased long-term care demands [1,2,4-7].

Botulinum toxin type A (BoNT-A), a potent neurotoxin, has demonstrated clinical efficacy across a range of neuromuscular disorders, including focal spasticity following stroke [8-13]. Its mechanism of action involves the inhibition of acetylcholine release at cholinergic nerve terminals within the neuromuscular junction, thereby interrupting synaptic transmission and resulting in dose-dependent, reversible muscle relaxation [10-12,14-16].

In the post-stroke population, focal spasticity frequently affects multiple muscle groups, leading to functional limitations and reduced quality of life [17-24]. Targeted intramuscular administration of botulinum toxin allows for localized modulation of spasticity, improving precision in therapeutic intervention and optimizing clinical outcomes [23,25-31].

Clinical benefits of BoNT-A treatment for post-stroke focal spasticity include improved motor function, increased joint range of motion, and reductions in pain and muscle overactivity [4,12,16,28,32,33]. Given its reversible nature, BoNT-A treatment regimens can be adjusted based on evolving patient needs, offering a flexible and patient-centred approach [21,25,29,34-36]. Importantly, BoNT-A therapy should be integrated into a comprehensive rehabilitation framework that includes encompassing physical therapy, occupational therapy, psychological support, and speech-language therapy, in order to maximize functional recovery and enhance overall quality of life [17,22,36,37,38].

### General guidelines for the functional assessment application

1. Safety considerations: ensure that assessments are conducted in a safe, controlled environment to minimize the risk of falls or injury.
2. Exclusion criteria: consider excluding patients with low Mini-Mental State Examination (MMSE) scores, aphasia, poor compliance, or comorbidities that pose a risk during testing.
3. Inclusion criteria: select patients based on the nature and severity of their neurological condition requiring functional evaluation.
4. Patient orientation: provide clear instructions and demonstrations to ensure patients understand the test procedures.
5. Encouragement: offer support and motivation throughout the evaluation to reduce frustration and promote optimal performance.
6. Setting and materials: assessments should be performed in appropriately equipped facilities with standardized materials to ensure consistency and accuracy.

It is essential to tailor the choice and application of assessment tools to each patient’s functional capabilities while adhering to standardized testing protocols [32,39-42].

### Functional assessment tools

A range of standardized instruments is employed to evaluate post-stroke functional status, each addressing specific aspects of motor performance, independence, and recovery. Ultimately, the selection and implementation of these tools should be tailored to the individual’s functional level while adhering to established clinical guidelines and protocols.

*The Modified Ashworth Scale (MAS)* is commonly used to quantify muscle tone by assessing resistance encountered during passive movement of a limb. The scale includes six grades, beginning with grade 0, indicating no increase in muscle tone, and progressing through grades 1 and 1+, which reflect slight increases in tone, with grade 1+ describing resistance through less than half of the range of motion. Grade 2 signifies a more marked increase in tone throughout most of the range, though the limb remains mobile. Grade 3 represents considerable resistance, making passive movement difficult, while grade 4 indicates that the limb is rigid in flexion or extension [43-45].

*Barthel Index (BI)* measures a patient's degree of independence in performing essential activities of daily living, such as feeding, bathing, dressing, managing bladder and bowel functions, transferring, ambulating, and stair climbing. This index is extensively validated in stroke populations and has also been applied to patients with neuromuscular and oncological conditions. Scores range from 0 to 100, with higher values corresponding to greater functional autonomy [42,46,47].

*Action Research Arm Test (ARAT)* is an observational tool designed to evaluate upper limb functionality, with a specific focus on grasp, grip, pinch, and gross motor movement. Each task is rated on a scale from 0 (inability to perform) to 3 (normal performance), with higher composite scores indicating better functional capacity. The ARAT has been validated for use in individuals with stroke, multiple sclerosis, Parkinson’s disease, and brachial plexus injuries. In terms of prognostic interpretation, scores below 10 suggest poor recovery potential, scores between 10 and 56 reflect moderate recovery, and scores of 57 and above indicate good functional prognosis [40,48-50].

*The Berg Balance Scale (BBS)* evaluates a patient’s balance through a series of 14 tasks that assess both static and dynamic stability, including activities such as standing, turning, and reaching. Each task is scored from 0 to 4, yielding a maximum score of 56 points. Lower scores are associated with a higher risk of falls and functional instability [51-53].

*Chedoke-McMaster Stroke Assessment (CMSA)* provides a structured approach to measuring physical recovery post-stroke. It consists of two major components: the Impairment Inventory, which assesses postural control, shoulder pain, and motor recovery stages for the arm, hand, leg, and foot on a scale of 1 to 7; and the Activity Inventory, which includes 10 items measuring gross motor function such as rolling, sitting, transferring, and standing, along with five items that evaluate walking capacity, also scored from 1 to 7 [54].

*Fugl-Meyer Assessment (FMA)* is a stroke-specific scale that evaluates five key domains: motor function, sensory function, balance, joint range of motion, and joint pain. It includes assessments of both the upper and lower extremities, with particular attention to movement quality and sensory feedback, such as light touch and joint position sense. The FMA is widely used in clinical practice and research as a tool for gauging impairment severity and guiding rehabilitation planning [49,55-58].

*Reintegration to Normal Living Index (RNLI)* is a self-reported measure designed to assess an individual’s ability to resume social, familial, and community roles following a disabling condition. Comprising 11 items rated on a 10-point scale, the RNLI captures the subjective experience of physical, emotional, and social reintegration into daily life [59,60].

Effective spasticity management requires a combination of medical treatment and rehabilitative intervention, with botulinum toxin serving as a key pharmacologic option [17,18,32,61,62]. This report aimed to evaluate the stage-dependent clinical outcomes of BoNT-A therapy in post-stroke spasticity using validated functional scales, with the hypothesis that earlier intervention yields superior functional gains.

## Material and Methods

### Study design

This CARE-compliant [63] report of four clinical cases was conducted in the specialized neurorehabilitation clinic of the National Institute for Medical Rehabilitation in Bucharest between January 2019 and December 2024.

### Patient selection

Participants were selected through consecutive convenience sampling from the institute’s inpatient population and were specifically selected to represent a range of stroke evolution stages (acute, subacute, and chronic), degrees of spasticity, as assessed using the Modified Ashworth Scale, and baseline motor control, allowing for comparative insight into variable clinical trajectories and treatment responsiveness. Eligible individuals presented a diagnosis of stroke—either ischemic or haemorrhagic—confirmed by computed tomography or magnetic resonance imaging, and clinically significant spasticity.

### Inclusion criteria

1. Patients who completed the written informed consent.
2. Age ≥ 18 and ≤ 80 years.
3. Confirmed diagnosis of stroke by CT or MRI.
4. Presence of clinically significant spasticity (Modified Ashworth Scale score ≥1+ and <4).
5. Completion of at least two inpatient rehabilitation programs, each consisting of a 15-day course of therapy (including physical therapy, physiotherapy, adjunctive methods, and orthotic use).

### Exclusion criteria

1. Patients with documented or reported medical history of adverse effects following previous BoNT-A injection (e.g., myalgia, muscle weakness, asthenia, flu-like syndrome, local reactions at the injection site, etc.).
2. Severe cognitive impairment.
3. Severe aphasia interfering with the patient’s assessment.
4. Degree of spasticity 1 or 4 on MAS (Modified Ashworth Scale).
5. Patients who refused the written informed consent.

### Assessment and procedure

Each patient completed two inpatient rehabilitation programs, consisting of a standardized multimodal rehabilitation program lasting 15 days each, and BoNT-A treatment. Follow-up was carried out 3 to 5 months after BoNT-A treatment. All interventions were supervised by a multidisciplinary team consisting of rehabilitation physicians, physiotherapists, occupational therapists, a speech and language therapist, and a clinical psychologist.

BoNT-A treatment was performed using abobotulinumtoxinA (Dysport), diluted to a concentration of 500 units in 2.5 to 5 mL of sterile saline solution. Injections were administered intramuscularly under ultrasound guidance, targeting muscles identified through clinical examination, palpation, and passive stretch testing. Doses were individualized according to muscle mass, degree of spasticity, and functional objectives, ranging from 35 to 300 units per muscle, with total session doses between 370 and 1200 units. Intervals between sessions varied from 12 to 20 weeks, depending on the patient’s clinical evolution and MAS score. Following each injection, patients performed passive stretching during the first 24 hours, followed by the gradual introduction of active exercises under physiotherapist supervision. (Table 1)

**Table 1:** Case summary table

Case	Stroke Type & stage	MAS range	BoNT-A doses (Total)	Injection sites	Main functional gains
Case 1	Hemorrhagic, Chronic	UL: 2–3, LL: 2	Up to 1200 U/session	Pectoralis, biceps, wrist/finger flexors, gastrocnemius, soleus, tibialis post.	Pain relief after the third injection; limited motor progress
Case 2	Hemorrhagic, Chronic	UL: 1+–2, LL: 1–2	Up to 650 U/session	Pectoralis, biceps, pronator teres, finger/wrist flexors, gastrocnemius, tibialis post.	Improved ADL independence, hand function, and balance
Case 3	Ischemic, Acute	UL: 2–3, LL: 1	Up to 370 U/session	Biceps, pronator teres, brachioradialis, finger/wrist flexors, pectoralis	Full ADL independence, gait recovery, social reintegration
Case 4	Hemorrhagic, Subacute	UL: 2–3, LL: 1+	Up to 720 U/session	Biceps, triceps, wrist/finger flexors, pronator teres	Improved balance, pain relief, and upper limb mobility

All patients participated in a standardized multimodal rehabilitation program throughout the study period. The program consisted of daily physiotherapy sessions of 45 to 60 minutes focusing on strength, balance, and task-specific motor training, as well as occupational therapy to retrain fine motor skills and activities of daily living. Adjunctive modalities such as electrostimulation, orthoses, or splints were applied as needed. Psychological support, speech and language therapy, and patient education were also integrated to optimize recovery and adherence.

Outcome assessments were conducted by an experienced physiatrist not involved in BoNT-A administration, both at baseline and three to five months after treatment. Spasticity was evaluated using the Modified Ashworth Scale. Functional independence was measured by the Barthel Index and Berg Balance Scale, while upper limb performance was assessed using the Action Research Arm Test and Fugl-Meyer Upper Extremity score. Global motor recovery was evaluated using the Chedoke-McMaster Stroke Assessment, and participation in daily and social activities was assessed with the Reintegration to Normal Living Index. Pain intensity was recorded using a 10-point Visual Analogue Scale. (Table 2)

**Table 2:** Functional outcomes summary

Case	Stroke stage	Barthel Index (Initial → Final)	Berg Balance Scale (Initial → Final)	ARAT score (Initial → Final)	Fugl-Meyer UE (Initial → Final)	RNLI (Initial → Final)	Pain reduction
Case 1	Chronic	65 → 75	14 → 24	0	11 → 15	45 → 59	After 3^rd^ injection
Case 2	Chronic	85 → 95	45 → 49	35 → 45	33 → 48	76 → 82	No pain initially
Case 3	Acute	80 → 100	46 → 54	42 → 51	—	74 → 102	Yes
Case 4	Subacute	55 → 95	7 → 50	—	—	40 → 84	Complete by 3 months

### Shoulder pain and functional limitation

This case involves a 66-year-old male patient who suffered a right capsulo-lenticular haemorrhagic stroke in June 2019, resulting in chronic left spastic hemiparesis. Given the time elapsed since the cerebrovascular event, the clinical condition was classified as chronic, requiring a long-term rehabilitation program and appropriate management of spasticity-related complications.

Baseline assessment performed in December 2021 revealed deficient motor control in the left upper limb, with an MRC score of 2/5 for the proximal and intermediate segments and 1/5 for the distal segment. Spasticity was present, graded 2 MAS for the pectoralis major and grade 3 for the flexor muscles of the elbow, wrist, and fingers. The patient reported both mechanical and neuropathic pain affecting the joints of the left limbs, predominantly at the shoulder, elbow, hip, and ankle. In the left lower limb, motor control was rated 3/5 proximally and 1/5 distally on the MRC scale. Tendon retractions of the Achilles tendon were also observed and confirmed by ultrasound evaluation, along with periarticular calcifications in the shoulder, elbow, and ankle joints. For support and proper positioning, a fixed wrist–hand orthosis and a fixed ankle–foot orthosis were used during ambulation.

The first administration of BoNT-A took place in December 2021. In the upper limb, 150 units were injected into the pectoralis major, brachialis, brachioradialis, superficial and deep flexor muscles of the fingers; 200 units into the flexor carpi radialis and pronator teres; and 300 units into the biceps brachii. In the lower limb, 150 units were injected into the medial and lateral gastrocnemius and the posterior tibialis, and 200 units into the soleus muscle.

A second injection was administered in April 2022, following a similar dosing scheme but yielding no notable improvement. A third injection was performed in July 2022, consisting of 100 units injected into the brachialis, flexor digitorum profundus, and flexor carpi radialis; 150 units into the flexor digitorum superficialis and pronator teres; and 200 units into the biceps brachii. For the lower limb, 100 units were injected into each of the medial and lateral gastrocnemius, soleus, and posterior tibialis muscles.

### Spasticity of the pronator teres and elbow flexors

This case concerns a 56-year-old female patient in the late chronic phase of post-stroke recovery, with a history of anterior communicating artery (ACA) aneurysm rupture in 2011, which resulted in right-sided hemiparesis and partially resolved expressive aphasia. Since the cerebrovascular event, the patient has consistently engaged in rehabilitation treatments and home-based physical therapy exercises. For upper limb positioning, she used a fixed wrist–hand orthosis, and for ambulation, a fixed ankle–foot orthosis on the right side. Notably, the patient had not previously received BoNT-A therapy.

Clinical examination revealed right upper limb motor control graded 4/5 MRC for proximal and intermediate segments, and 3/5 for distal segments. Spasticity was recorded as grade 1+ MAS for the pectoralis major and grade 2 for the flexor muscles of the elbow, wrist, and fingers. No pain or tendon retractions were observed. In the lower limb, motor control was preserved and rated 5/5 MRC for proximal and intermediate segments, and 3/5 for the distal segment. Spasticity was mild, graded 1 MAS for the adductor muscles, 1+ for the quadriceps, and 2 for the gastrocnemius, soleus, and posterior tibialis muscles, again without evidence of retraction or pain.

The first injection of BoNT-A included 50 units in the brachioradialis, deep finger flexors, and the long and short flexors of the thumb; 100 units in the pectoralis major, biceps brachii, brachialis, pronator teres, and flexor carpi radialis; and 150 units in the superficial finger flexors. In the lower limb, 50 units were injected into the posterior tibialis and 100 units each into the medial and lateral gastrocnemius and soleus muscles. A second injection was performed 4 months later, using a slightly reduced dosage (approximately 20 units less per injected muscle), targeting the same muscle groups.

### Clenched fist and hand dysfunction

The third case concerns a 56-year-old male patient in the acute stage of post-stroke recovery, following a right sylvian ischemic stroke that occurred in August 2020, which resulted in left-sided spastic hemiparesis. As part of his rehabilitation management, the patient used a fixed wrist–hand orthosis designed to ensure proper positioning of the affected upper limb. The patient also developed a secondary depressive episode, attributed to the sudden loss of functional independence and the inability to perform daily activities.

Clinical evaluation revealed relatively preserved motor control in the left upper limb, scoring 4−/5 to 4/5 MRC for the proximal and intermediate muscle groups, and 3/5 for the distal segments. However, moderate to severe spasticity was noted, graded 2–3 MAS, affecting the flexor muscles of the elbow, wrist, and fingers. In the left lower limb, motor control was preserved across all segments, scoring 4/5 MRC for proximal, intermediate, and distal muscle groups. Spasticity was mild, graded 1+ MAS, involving the plantar flexor muscles. No tendon retractions or pain were observed in either limb.

The first injection of BoNT-A was performed 25 days after stroke onset, which included the injection of 50 units into the brachioradialis and flexor carpi radialis muscles, and 100 units each into the biceps brachii, flexor digitorum superficialis, and pronator teres muscles.

A second BoNT-A injection was administered five months later, with adjusted dosing: 50 units into the pronator teres muscle, 70 units into the flexor digitorum superficialis muscle, and 100 units each into the biceps brachii and pectoralis major muscles.

### Combined flexor and extensor spasticity of the elbow

The fourth case describes a 37-year-old female patient in the subacute phase of recovery following a haemorrhagic stroke in June 2021, which resulted in left-sided spastic hemiparesis. Initially, in August 2021, the patient required assistance for all types of transfers and was unable to ambulate. However, by February 2022, her clinical condition had improved significantly—she became able to perform transfers independently and ambulate using a tripod cane and a fixed ankle–foot orthosis on the left side. This favorable evolution during the subacute period reflects a positive response to early rehabilitation interventions and supports the importance of continued therapy to promote functional recovery further.

Clinical examination of the left upper limb revealed motor deficits, with muscle strength rated 3/5 for proximal segments, 2/5 for intermediate segments, and 1/5 MRC for distal segments. Spasticity was clinically evident, particularly in the triceps brachii muscle (grade 2–3 MAS), as well as in the flexor muscles of the elbow, wrist, and fingers, where it was rated 2 MAS. The patient also reported neuropathic pain in the affected upper limb, predominantly in proximal regions. In the left lower limb, motor control was comparatively better, with all proximal, intermediate, and distal muscle groups rated between 4−/5 and 4/5 MRC. Spasticity of the plantar flexors was mild (MAS grade 1+), and no tendon retractions were observed in either limb.

The patient received two BoNT-A injection sessions targeting the spastic left upper limb. The first was administered two months post-stroke and included the following muscles: biceps brachii, brachialis, flexor carpi radialis and ulnaris, pronator teres, deep and superficial finger flexors, and triceps brachii. Doses ranged from 35 to 150 units per muscle group.

A second injection was administered five months later, targeting the biceps brachii, triceps brachii, pronator teres, and flexor digitorum superficialis muscles, with adjusted—progressively reduced—doses to optimize therapeutic response.

## Results

### Case 1 (chronic stage): modest functional progress

- Barthel Index: 65 → 75
- Berg Balance Scale: 14 → 24
- ARAT: constant score of 0
- FM-UE: 11 → 15
- RNLI: 45 → 59
- Neuropathic pain improved after the third injection → weak and delayed therapeutic response

**Figure 1:**
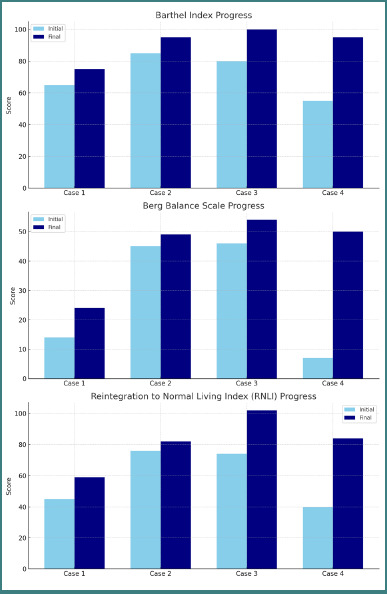
Progress of BI, BBS, RNLI

**Figure 2:**
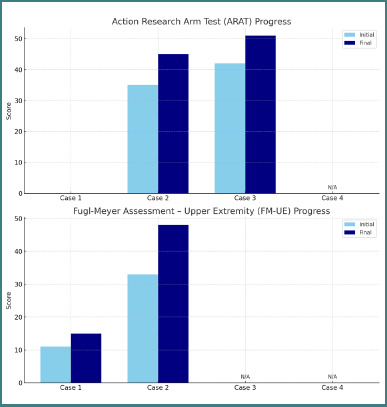
Progress of ARAT, FM-UE

The patient, in the chronic phase of recovery, showed a modest increase in the Barthel Index from 65 to 75 and in the BBS score from 14 to 24, indicating limited functional gains with subsequent plateauing of progress (Figure 1). The ARAT score remained at 0, while the FM-UE score rose slightly from 11 to 15, suggesting restricted motor recovery (Figure 2). RNLI improved marginally from 45 to 59. Neuropathic pain showed partial relief after the third BoNT-A administration, indicating a delayed and suboptimal therapeutic response.

### Case 2 (late chronic stage): slow but favourable response

- Barthel Index: 85 → 95
- Berg Balance Scale: 45 → 49
- ARAT: 35 → 45
- FM-UE: 33 → 48
- RNLI: 76 → 82
- No pain

The patient, in the late chronic phase—ten years post-stroke—demonstrated a slow-to-moderate yet encouraging functional improvement, which was particularly noteworthy given the chronicity of the spasticity and the fact that this was her first administration of BoNT-A since the initial stroke.

Functional outcomes showed an increase in the Barthel Index from 85 to 95, BBS score from 45 to 49, the ARAT score from 35 to 45, and the FM-UE score from 33 to 48, indicating a meaningful functional enhancement of the upper limb. RNLI improved from 76 to 82, and no pain was reported either at baseline or during treatment.

Gait pattern became more stable, fall risk was minimal, and the patient no longer required orthoses or a cane. Additionally, she showed greater engagement in recreational and social activities and improvement in speech production—in the context of expressive aphasia—with progress in spontaneous speech and object naming. Voluntary upper limb movements improved both within and outside synergy patterns, with better coordination, speed, and functional use.

### Case 3 (acute stage): significant functional improvements

- Barthel Index: 80 → 100
- Berg Balance Scale: 46 → 54
- ARAT: 42 → 51
- RNLI: 74 → 102
- CMSA – CAHAI:
  - Arm function: 5/7 → 6/7
  - Hand function: 3/7 → 5/7
  - Lower limb: 7/7
  - Foot: 5/7 → 6/7
  - Gross motor function: maximum score 7/7 → total: 28/35 → 31/35
  - Walking Index: 92/100 → 96/100

The patient, in the acute stage of recovery, demonstrated remarkable functional progress. Barthel Index increased from 80 to 100, BBS score from 46 to 54, the ARAT score from 42 to 51, and RNLI showed a substantial improvement from 74 to 102. The CAHAI component of the CMSA indicated marked enhancement across multiple functional domains: arm function improved from 5/7 to 6/7, hand function from 3/7 to 5/7, lower limb function reached the maximum score of 7/7, and foot function rose from 5/7 to 6/7. The gross motor function index achieved the maximum score of 7/7. These findings correspond with improvements in voluntary movement control and balance, as well as enhancement of gait pattern, confirmed by the Walking Index increase from 92/100 to 96/100.

Functionally, the patient exhibited significant improvements in mobility, coordination, and voluntary motor control of the left upper limb, accompanied by reduced spasticity. Gait quality improved notably, and final balance scores indicated a minimal risk of falls, even under conditions of fatigue or uneven terrain.

A remarkable aspect of this case was the patient’s successful return to professional activity as a photographer, along with reintegration into recreational, social, and family life. Pain levels decreased over the course of treatment, and the patient’s overall psychological state also improved considerably, contributing to the favourable functional outcome.

### Case 4 (subacute stage): significant favourable changes

- Barthel Index: 55 → 95
- Berg Balance Scale: 7 → 50
- RNLI: 40 → 84
- CMSA – CAHAI:
  - Arm function: 4/7 → 6/7
  - Hand function: 2/7 → 5/7
  - Lower limb: 6/7
  - Foot: 5/7 → 6/7
  - Gross motor function: maximum score 7/7 → total: 23/35 → 30/35
  - Walking Index: 87/100 → 93/100
- Complete pain remission within 3 months post-injection
- Marked improvement in mobility and balance

The patient, in the subacute stage of recovery, exhibited some of the most striking improvements observed in the series. Barthel Index increased from 55 to 95, and BBS score from 7 to 50, indicating a major leap in mobility and balance. According to CMSA, the CAHAI component improved from 23/35 to 30/35, while the Walking Index rose from 87/100 to 93/100, demonstrating substantial enhancement in upper limb coordination, static and dynamic balance, and gait pattern. RNLI increased significantly, from 40 to 84. Pain symptoms resolved completely within three months following the initial BoNT-A injection.

## Discussion

Botulinum toxin type A plays a pivotal role in the multidisciplinary management of post-stroke spasticity. Its therapeutic effects extend beyond peripheral muscle relaxation to include central neuromodulatory mechanisms that contribute to improved motor control and pain reduction.

### Peripheral effects and role in preventing maladaptive changes

Peripherally, BoNT-A acts by reducing muscle hyperactivity and preventing secondary musculoskeletal complications such as tendon contractures and periarticular heterotopic ossifications [18,64-66]. These effects are reinforced by concurrent use of stretching programs and orthotic interventions, which enhance proprioceptive feedback and promote optimal joint alignment, thus supporting the overall functional rehabilitation process. In cases of severe spasticity, maintaining motor function through this combined approach is crucial for preventing long-term deformities and improving comfort and daily activity performance [22,67].

### Central neuromodulatory effects

Beyond its local action, BoNT-A has been increasingly recognized for its central neuromodulatory effects [65,68-70]. By reducing abnormal muscle tone and suppressing involuntary motor activity, it modifies afferent proprioceptive input to the central nervous system, interrupting maladaptive sensorimotor loops and creating a more favorable environment for cortical reorganization and refinement of motor pathways. Decreasing hyperexcitability in descending tracts—particularly within the reticulospinal pathway—facilitates attenuation of pathological synergies and supports the reemergence of selective, physiologic movement patterns. These mechanisms are especially relevant in the early post-stroke period, when neural plasticity and responsiveness to intervention are at their peak.

Even in the chronic stage, BoNT-A remains valuable in promoting motor re-education and functional stabilization, particularly when integrated into task-specific rehabilitation programs. Functional neuroimaging studies have demonstrated increased activation in cortical areas involved in motor planning and sensory integration after BoNT-A treatment, reinforcing its role in facilitating adaptive neuroplasticity [31,70-74].

### Pain modulation and non-motor benefits

The therapeutic benefits of BoNT-A extend beyond improvements in tone and motor performance. Several studies have demonstrated its efficacy in the management of post-stroke neuropathic pain, particularly in cases of central post-stroke pain (CPSP), which often proves refractory to standard pharmacological therapies [69-71,75-77]. CPSP typically results from ischemic or haemorrhagic lesions involving the brainstem, thalamus, or cortex and manifests through persistent pain and sensory abnormalities. Experimental evidence suggests that peripherally administered BoNT-A may undergo retrograde axonal transport and modulate central synaptic activity, contributing to its analgesic properties. A double-blind study [75] reported significant and sustained reductions in pain—lasting up to 6 months—in patients receiving 500 units of BoNT-A for upper limb spasticity.

### Functional assessment and individualized rehabilitation

Accurate evaluation and individualized treatment planning are essential to achieve optimal outcomes. The use of standardized functional assessment tools enables clinicians to monitor therapeutic efficacy and adapt rehabilitation strategies to each patient’s specific needs. For patients with mild to moderate spasticity and preserved motor control, instruments such as the Chedoke-McMaster Stroke Assessment (CMSA), Fugl-Meyer Assessment (FMA), and Action Research Arm Test (ARAT) provided detailed, sensitive insights into motor function, coordination, and task execution. In cases of severe spasticity or advanced impairment, targeted subscales such as the FMA Upper Extremity (FM-UE) and CMSA allow a comprehensive appraisal of both motor and sensory domains, including passive mobility and pain. Selecting assessment tools according to recovery stage and functional profile enables dynamic tracking of progress and precise redefinition of therapeutic objectives [21,62].

In this report, patients with moderate or mild spasticity who retained partial motor control demonstrated notable functional improvement, supported by the use of progressively reduced BoNT-A doses and extended injection intervals beyond three months. These outcomes suggest a favorable therapeutic response and the persistence of motor plasticity [78]. Instruments such as the CMSA (CAHAI and Walking Index components) and FM-UE proved particularly effective for detecting incremental changes in coordination, upper limb dexterity, and gait function.

The Reintegration to Normal Living Index (RNLI) provides valuable insights into how patients perceive their own progress beyond strictly functional outcomes [8,11,17]. By capturing psychological well-being, emotional adaptation, and the individual’s sense of participation and autonomy, the RNLI enables clinicians to tailor rehabilitation goals to what truly matters to each patient. Incorporating this broader, patient-centered perspective helps optimize therapeutic management, ensuring that treatment aligns not only with measurable physical recovery but also with the individual’s personal and social reintegration trajectory.

## Conclusion

This study highlights the therapeutic value of BoNT-A across all stages of post-stroke recovery, underscoring the need for a stage-specific, patient-centered rehabilitation strategy. The effectiveness of BoNT-A depends on the timing of intervention, the severity and distribution of spasticity, and the individual’s baseline motor control. In the acute and subacute phases, early BoNT-A administration was associated with improved voluntary motor control, enhanced engagement in physiotherapy and occupational therapy, and a reduction in secondary complications such as contractures, leading to greater functional independence. In the chronic stage, BoNT-A contributed to maintaining motor function, preventing musculoskeletal complications, and alleviating neuropathic pain, remaining clinically valuable in preserving mobility and quality of life. Standardized assessment tools allow clinicians to monitor progress objectively and tailor therapy to each patient’s motor status and functional potential. The Fugl–Meyer Assessment and Fugl–Meyer Upper Extremity scale are more useful during the acute and subacute stages, when early motor recovery and neuroplasticity are most active. In the subacute to early chronic phases, the Chedoke–McMaster Stroke Assessment and its components (such as CAHAI and the Walking Index) are better suited to evaluate coordination and task-specific performance. For the chronic stage, when rehabilitation focuses on independence and participation, instruments like the Action Research Arm Test, Barthel Index, Berg Balance Scale, and Reintegration to Normal Living Index are valuable for assessing adaptive function and perceived quality of life. An important contribution to the various degrees of effectiveness of BoNT-A therapy across the four cases was closely linked to the earnest striving of the clinical team to align therapeutic goals with appropriate evaluation tools, calibrating target selection, dosage, and injection frequency to the patient’s recovery potential. Thus, when integrated into a multidisciplinary rehabilitation framework—combining medical, physical, occupational, and psychological interventions—BoNT-A supported functional recovery, reduced the burden of spasticity, and improved overall quality of life, reinstating that it is an effective and adaptable intervention throughout the continuum of post-stroke rehabilitation, offering its greatest benefits when applied early and guided by individualized, functionally driven assessment.
